# miR-202 inhibits the progression of human cervical cancer through inhibition of cyclin D1

**DOI:** 10.18632/oncotarget.12499

**Published:** 2016-10-06

**Authors:** Yuexiong Yi, Huirong Li, Qiongying Lv, Kejia Wu, Wenfen Zhang, Juan Zhang, Dingjun Zhu, Qing Liu, Wei Zhang

**Affiliations:** ^1^ Department of Obstetrics and Gynecology, Zhongnan Hospital of Wuhan University, Wuhan University, Wuhan 430071, P.R. China; ^2^ Department of Gynecology, Shandong Jiaohong Hospital, Jinan, Shandong 250031, P.R. China; ^3^ Department of Gynecology, The 5th Hospital of Jinan, Shandong 250031, P.R. China; ^4^ The First Department of Gynecology, Renmin Hospital of Wuhan University, Wuhan University, Wuhan 430071, P.R. China; ^5^ Department of Gynecology, Shiyan Materal and Children Health Hospital, Shiyan 44200, P.R. China

**Keywords:** miR-202, cyclin D1, cervical cancer

## Abstract

The human cervical cancer (CC) acts as the most common one of women tumors. However, the pathological changes and molecular alterations of CC are not clear. It has been reported that miR-202 takes part in the development and progression of different tumors. The present study aims to detect the expression of miR-202 in 100 cases of CC tissues and cells, and then we continued to investigate the potential mechanisms of miR-202 in CC cells. In this work, we found that the expression of miR-202 is obviously decreased in both CC cell lines and tissues, and negatively related with the expression of cyclin D1 in SiHa, HeLa and Caski cells. *In-vitro* assay revealed that the ectopic expression of miR-202 suppressed the proliferation, migration and invasion of SiHa and HeLa cells. Additionally, the over-expression of miR-202 extremely affected the expression of cyclin D1 protein. Notably, the over-expression of cyclin D1 in SiHa and HeLa cells with miR-202 mimics attenuated the inhibitory effects of miR-202 on cell proliferation, migration and invasion. In conclusion, our study identified that miR-202 plays an important role in regulating cell proliferation, migration and invasion of CC by directly targeting cyclin D1, thus miR-202 may represent a potential therapeutic target for patients with cervical cancer.

## INTRODUCTION

Cervical cancer act as the second most common tumor among women in this world [1, 2]. It is estimated that about five millions of women patients are newly diagnosed and approximately two millions of cases died of cervical cancer every year. Accumulating evidence showed that human papillomavirus (oncogenic types) plays a crucial role in the induction and development of human cervical cancer [3–5]. As reported, some patients did not infect human papillomavirus, suggesting that more potential factors promote the malignant progression of cervical cancer [6–7]. Although some related studies were conducted, the molecular etiology of cervical cancer is still largely unknown. In the present study, we will investigate the molecular mechanisms implicated in the development of cervical cancer.

MiRNAs are a group of small single-stranded non-coding RNAs with a length of approximately 22 nucleotides. MiRNAs are involved and play critical roles in a variety of biological processes through suppressing the expression of target messenger RNAs [8–11]. The miRNAs have been identified to be involved in different cancer cell procession, which indicates cell proliferation, differentiation, migration and invasion [12–15]. Increasing reports have demonstrated that the alteration of miRNAs is related to the development of different tumors, such as pancreatic cancer, lung cancer, and breast cancer [16–18]. Accumulating studies showed that the expression of miR-202 was deregulated in different cancers, including colorectal carcinoma [19], gastric cancer [20], myeloma [21], osteosarcoma and lung carcinoma [22, 23]. Recently miR-202 was reported as a tumor suppressor miRNA. For instance, down-regulated miR-202 regulates Mxd1 and Sin3A repressor complexes, by which miR-202 mediates the proliferation and apoptosis of pancreatic cancer cells. [18]. However, the expression and mechanism of miR-202 in cervical cancer remain to be unclear.

In this work, we explored the potential role of miR-202 in the progression of cervical cancer. Our findings revealed that the expression of miR-202 is remarkably down regulated in human CC samples. Afterwards, the mechanisms underlying the down-regulation of miR-202 were investigated in the development of CC. The outcome suggested that cyclin D1 is a downstream target of miR-202, and miR-202 plays a crucial role in CC cell proliferation, migration and invasion by directly regulating cyclin D1.

## RESULTS

### The expression of miR-202 is decreased in CC cell lines and samples

**Figure 1:**
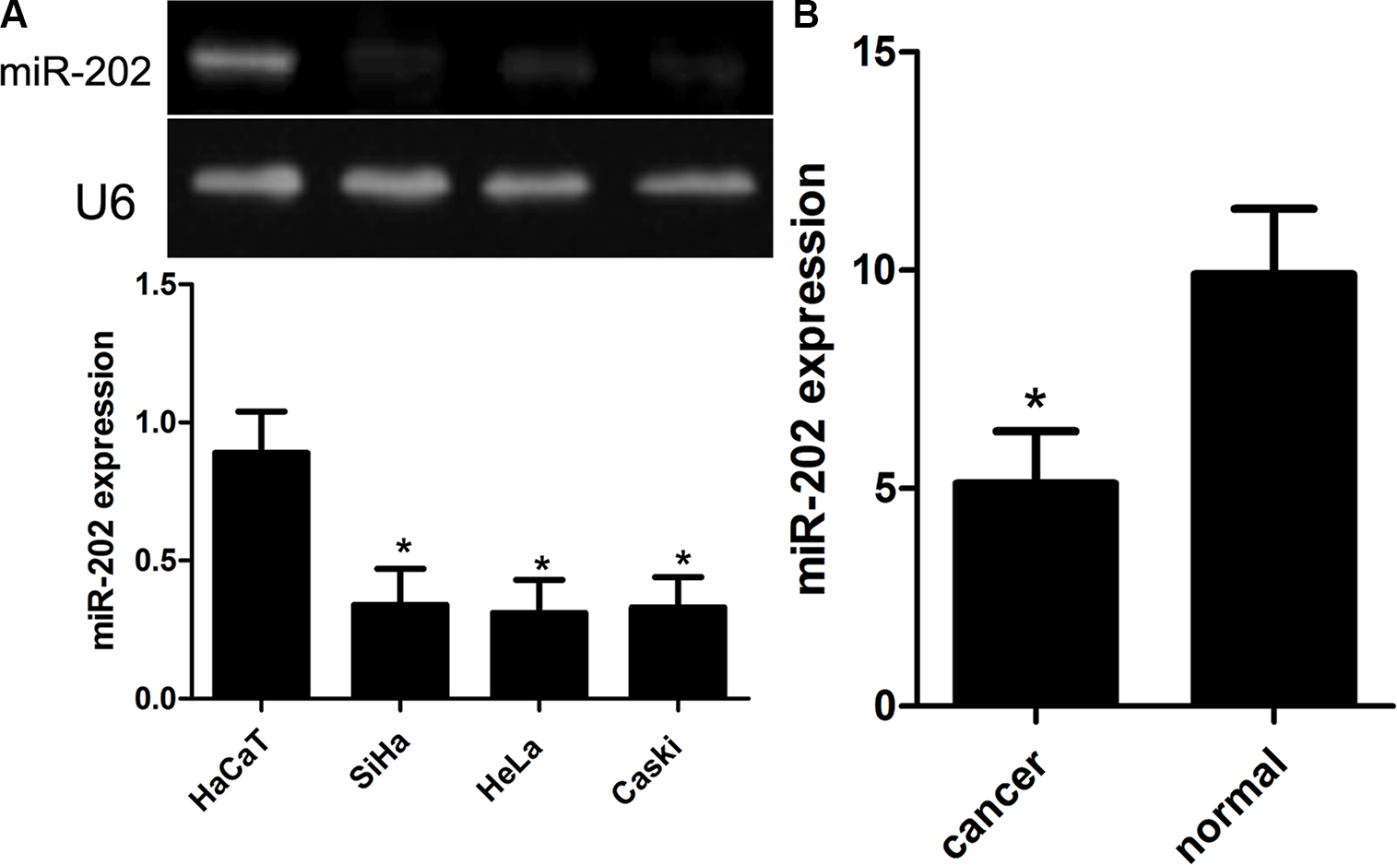
Reduced miR-202 expression in CC cell lines and tissues (**A**) Relative miR-202 expression in CC cell lines (SiHa, HeLa, and Caski) and human non-tumor keratinocyte line HaCaT. (**B**) Relative miR-202 expression in 100 pairs of CC tissues and adjacent normal counterpart tissues was detected using real-time RT-PCR. *p < 0.001, vs HaCaT or normal tissues.

To figure out the significance of miR-202 expression in the development of CC, firstly, the expression of miR-202 was detected using qRT-PCR and RT-PCR in CC cell lines, including SiHa, HeLa, and Caski, and human non-tumor keratinocyte line HaCaT as well as CC samples. In this wok, we found that SiHa, HeLa, and Caski cell lines had significantly lower expression of miR-202 compared with normal HaCaT cells (Figure 1A). Among 100 cases of CC samples, the expression of miR-202 was obviously down-regulated in 68 cases of samples. And moreover, the expression of miR-202 was obviously up-regulated in all adjacent normal cervical tissues (Figure 1B). In addition, the non-parametric test U Mann Whitney revealed that the down-regulation of miR-202 was closely related to the grade (*p* = 0.001) and stage (*p* = 0.002) of cervical cancer. At the same time, we also found that 68 patients with low miR-202 expression have shorter survival period compared with the rest with high miR-202 expression using the survival curve and log-rank test (*p* = 0.012).

### miR-202 affects the cell proliferation, migration and invasion

**Figure 2:**
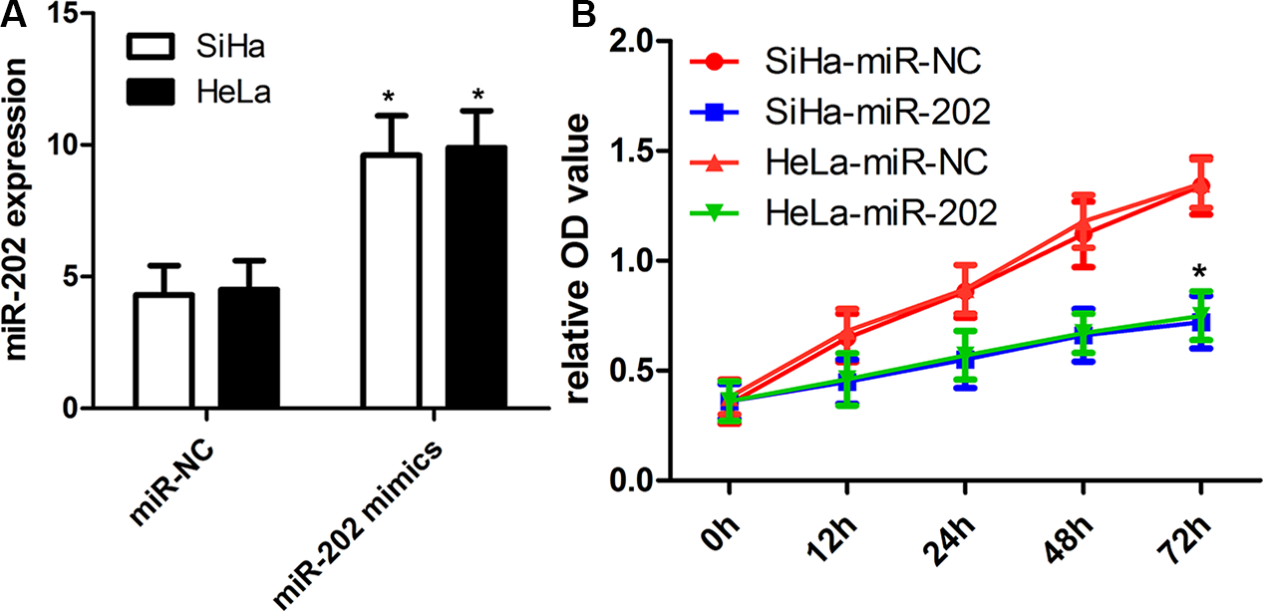
miR-202 inhibits CC cell proliferation (**A**) Relative miR-202 expression in SiHa and HeLa cells was measured after the cells were transfected with miR-202 mimics or scramble control miRNA using real-time RT-PCR. (**B**) Cell proliferation was measured using a CCK-8 assay after 24 hours transfection. SiHa and HeLa cells were transfected with miR-202 mimics or scramble control miRNA. *p < 0.001, vs control.

**Figure 3:**
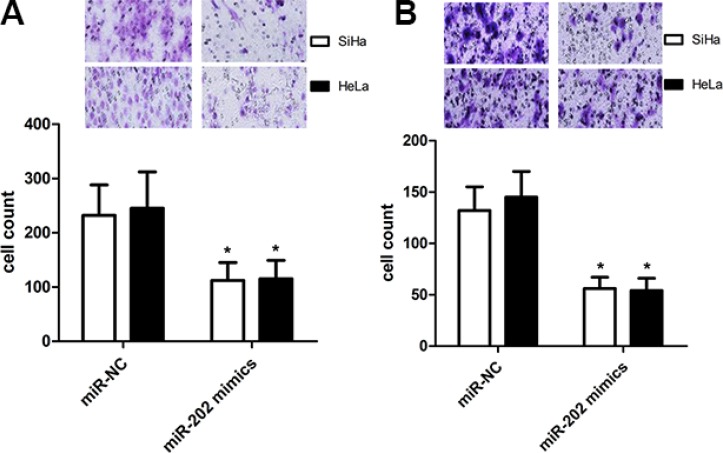
miR-202 inhibits CC cell migration and invasion (**A**) The migration capacity of SiHa and HeLa cells was measured by transwell migration assay after transfecting the cells with miR-202 mimics or scramble control miRNA for 48 h. Overexpresion of miR-202 inhibited the invasion of SiHa cells. The relative ratio of invasive cells per field is shown. (**B**) The invasive capacity of SiHa and HeLa cells was assessed by transwell invasion assay after transfecting the cells with miR-202 mimics or scramble control miRNA for 48 h. Overexpresion of miR-202 inhibited the invasion of SiHa and HeLa cells. The relative ratio of invasive cells per field is shown. *p < 0.001, vs control.

To figure out the value of miR-202 in cell proliferation of cervical cancer, we applied miR-202 overexpressing SiHa and HeLa cells by transiently transfecting cells with miR-202 mimics. Firstly the overexpression of miR-202 was confirmed in SiHa and HeLa cells using qRT-PCR (Figure 2A). Secondly, overexpression of miR-202 led to obviously reduced proliferation capacity in both SiHa and HeLa cells (Figure 2B). To characterize the role of miR-202 in cell migration and invasion of cervical cancer, tranwell migration and invasion assay was carried out to evaluate the effects of miR-202 on the migration and invasion of SiHa and HeLa cells. The tranwell assay revealed that overexpression of miR-202 suppressed the migration and invasion of SiHa and HeLa cells compared with miR-NC control (Figure 3A, 3B).

### cyclin D1 is identified as a target of miR-202

**Figure 4:**
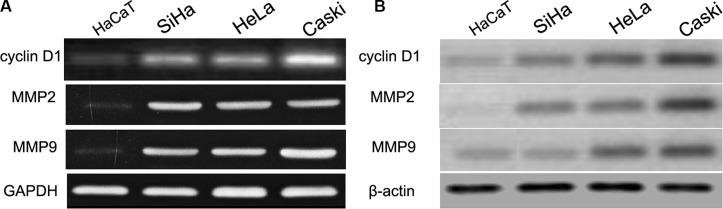
cyclin D1, MMP2 and MMP9 were up-regulated in CC cell lines and HaCaT cells The mRNA and protein level of cyclin D1, MMP2 and MMP9 was measured in CC cell lines using western blot. β-actin and GAPDH was used as an internal loading control. The mRNA and protein expression level was calculated using Image J Pro software. Each bar represents the mean ± SD of three independent experiments; *p < 0.001, compared with HaCaT.

**Figure 5:**
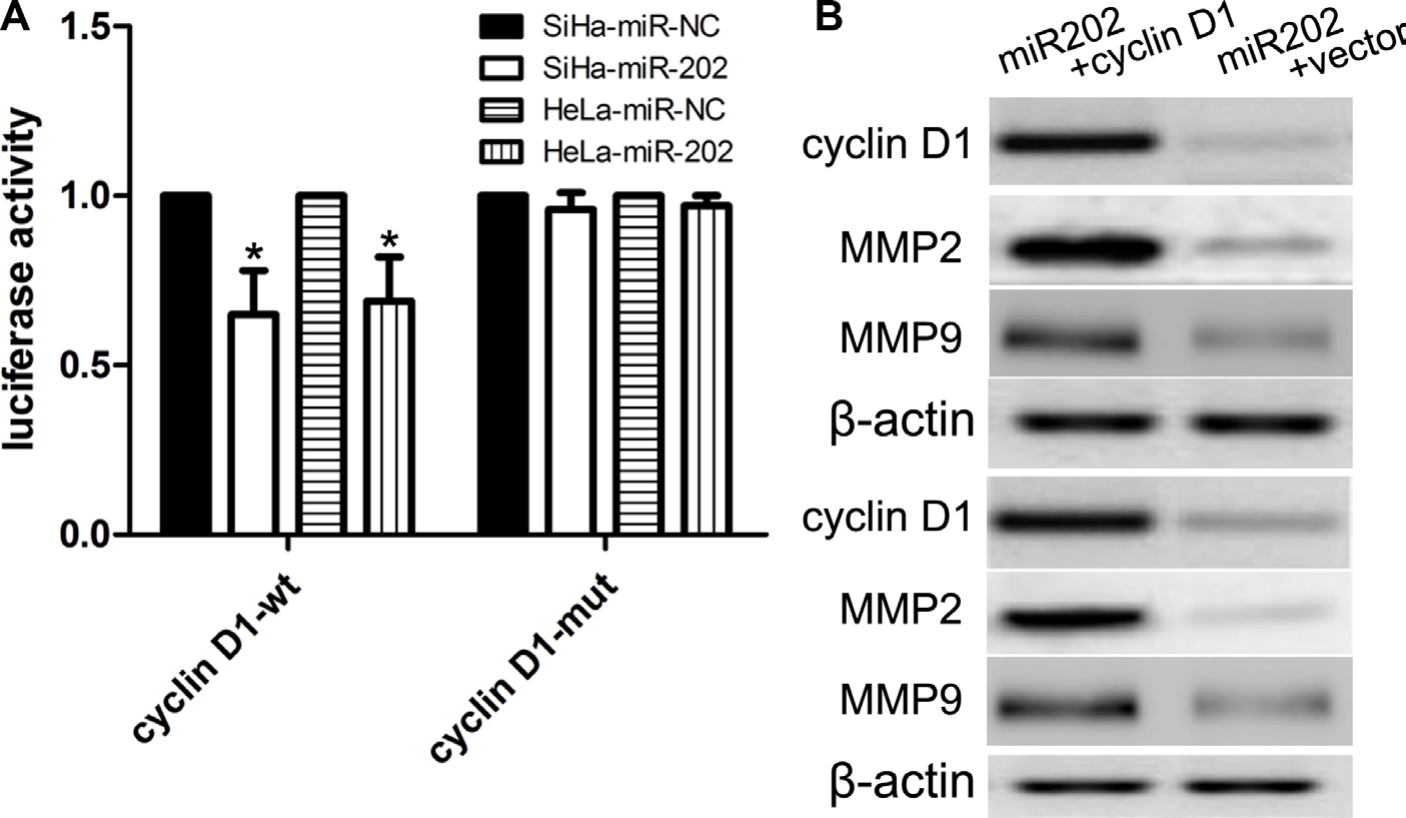
cyclin D1 is a candidate target of miR-202 (**A**) The miR-202 mimics inhibited the luciferase activity controlled by wild-type cyclin D1-3′-UTR, but did not affect the luciferase activity controlled by mutant cyclin D1-3′-UTR in SiHa cells. At the same time, miR-202 mimics inhibited the luciferase activity controlled by wild-type cyclin D1-3′-UTR, but did not affect the luciferase activity controlled by mutant cyclin D1-3′-UTR in HeLa cells. (**B**) Western blot analysis showed that transfecting the cells with cyclin D1 plasmids could up-regulate cyclin D1 expression. The expression of cyclin D1 was normalized to that of β-actin. *p < 0.001, vs. miR-NC control.

Previously published reports demonstrated that the cyclin D1 3′UTR can act as a putative miR-202 binding site [23]. In this work, we firstly detected the cyclin D1 expression using real time PCR and western blot in CC cell lines (SiHa, HeLa, and Caski) and HaCaT cells. We found that SiHa, HeLa, and Caski cells had the higher mRNA and protein expression of cyclin D1 than did the HaCaT cells (Figure 4A, 4B), indicating that SiHa, HeLa, and Caski cells have the stronger proliferation capacity. To further elucidate the capacity of migration and invasion of these cells, we detected the expression of MMP2 and MMP9, and found that the expression profile of MMP2 and MMP9 was consistent with cyclin D1. In addition, in comparison with cyclin D1 mutation-type 3′UTR, the luciferase reporter activity was decreased by approximately 40% in SiHa cells or 45% in HeLa cells containing the cyclin D1 wild-type 3′UTR fragment (Figure 5A). Besides, we made a correlation analysis to elucidate the negative association of miR-202 and cyclin D1 in SiHa, HeLa, and Caski cells. Finally, we analyze the correlation between cyclin D1 and miR-202 mRNA levels in 100 tumor samples and also found the negative association of miR-202 and cyclin D1 in the 100 tumor samples.

### Enforced cyclin D1 expression attenuates the inhibitory effects of miR-202

**Figure 6:**
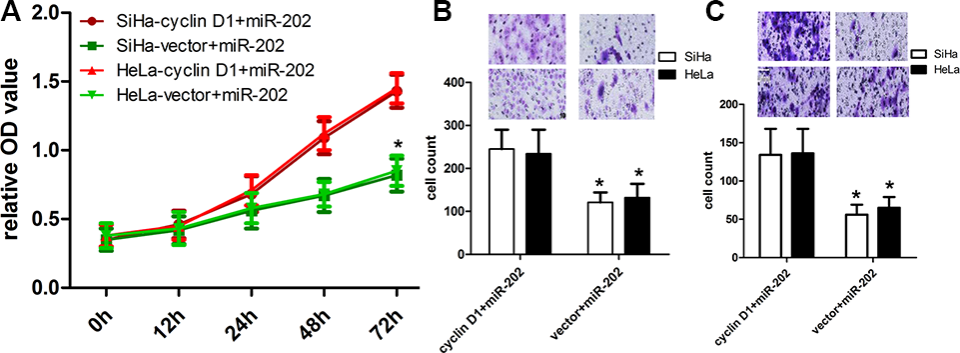
cyclin D1 overexpression reversed the inhibitory effect of miR-202 on CC cell proliferation, migration and invasion (**A**) The proliferation capacity of miR-202-overexpressing SiHa and HeLa cells was partially improved when cells were transfected with cyclin D1 plasmids in comparison with miR-NC. (**B**, **C**) The migration and invasion of miR-202-overexpressing SiHa and HeLa cells were effectively improved when cells were transfected with cyclin D1 plasmids. *p < 0.001, vs. vector.

To further figure out the relationships between miR-202 and cyclin D1, we transfected pcDNA3.1(+)-cyclin D1 plasmids into miR-202-overexpressing SiHa and HeLa cells to overexpress cyclin D1 protein (Figure 5B). The CCK-8 proliferation assay revealed that overexpression of cyclin D1 enforced the proliferation potentials of SiHa and HeLa cells (Figure 6A). In addition, the transwell assay also revealed that overexpression of cyclin D1 in miR-202-overexpressing SiHa and HeLa cells enhanced the migration and invasion capacity of SiHa and HeLa cells in comparison with vector control (Figure 6B, 6C).

### Inhibited cyclin D1 expression enhances the inhibitory effects of miR-202

**Figure 7:**
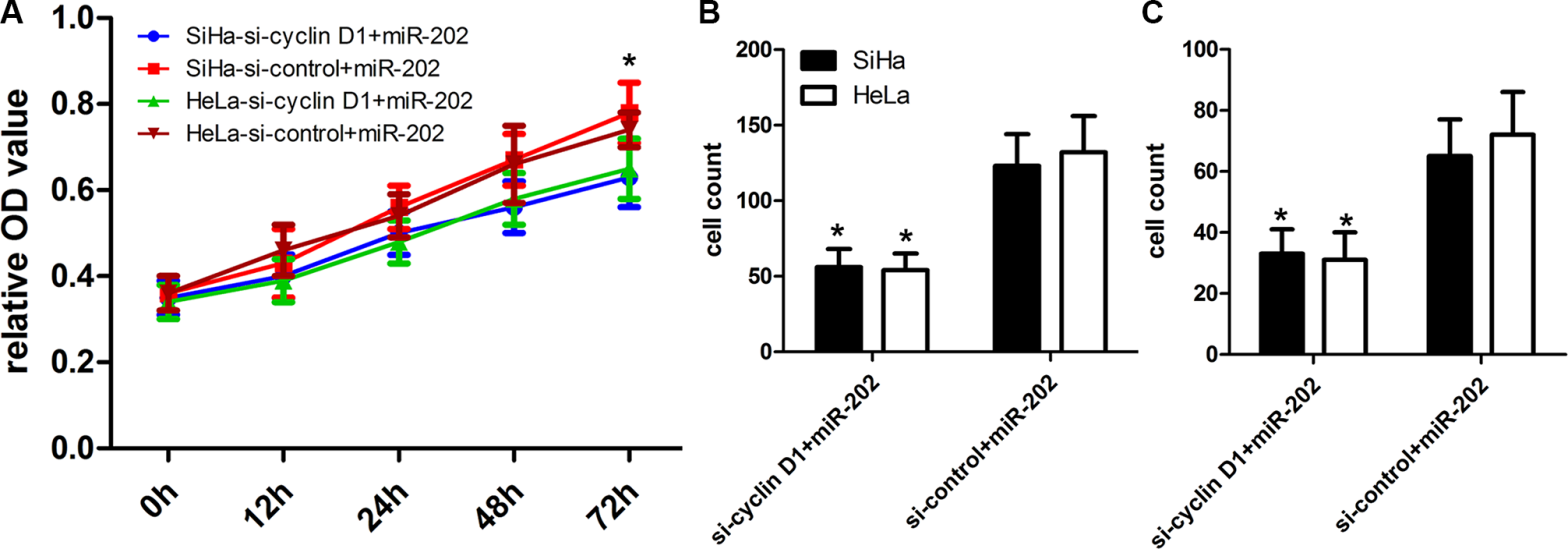
cyclin D1 inhibition enhanced the inhibitory effect of miR-202 on CC cell proliferation, migration and invasion (**A**) The proliferation capacity of miR-202-overexpressing SiHa and HeLa cells was partially inhibited when cells were transfected with si-cyclin D1 compared with si-control. (**B**, **C**) The migration and invasion of miR-202-overexpressing SiHa and HeLa cells were effectively improved when cells were transfected with si-cyclin D1. *p < 0.001, vs. si-control.

To further figure out the relationships between miR-202 and cyclin D1, we conducted a gene silencing assay, and transfected cyclin D1 siRNA and control siRNA into miR202-overexpressing SiHa and HeLa cells to inhibit the expression of cyclin D1 protein. The CCK-8 proliferation assay showed that the inhibited expression of cyclin D1 affected the proliferation potentials of SiHa and HeLa cells (Figure 7A). In addition, the transwell assay also showed that inhibited expression of cyclin D1 in miR-202-overexpressing SiHa and HeLa cells affected the migration and invasion potentials of SiHa and HeLa cells in comparison with si-control (Figure 7B, 7C).

## DISCUSSION

As is known to all, the family of D-type cyclins (D1-3) plays an important role in the regulation of the G1/S transition, among which D-type cyclins can bind and trigger the activity of cyclin-dependent kinases Cdk4 and Cdk6 to activate the activity of the retinoblastoma protein [24–26]. Notably, cyclin D1 is involved in some pathological and physiological processes, including the self-renewal of mammary epithelial cell, and the proliferation properties of tumors [27]. In this study, we examined the existence of miR-202-induced cyclin D1 pathway in CC, and elucidate the significance of miR-202-induced cyclin D1 pathway.

It has been reported that miR-202 was found to be decreased in some kinds of tumors. For example, miR-202 was found to be obviously altered in most of pancreatic cancer tissues. Recently, some reports showed that miR-202 acts as a tumor suppressor, and the expression of miR-202 is identified to be down-regulated in colorectal carcinoma [19], gastric cancer [20], myeloma [21]. Up to date, the expression and potential role of miR-202 in the development of CC is little known. In this study, our findings showed that miR-202 was down-regulated, and miR-202 was involved in the development of CC. Here we listed the potential reasons why miR-202 was down-regulated. Firstly, the generation of mature miRNA needs two basic processed: (1), the endogenous transcript pri-miRNA is cleavaged by Drosha enzyme to generate the 70 nucleotides pre-miRNA, which showed peculiar stem-loop structure in the cell nucleus; (2), After that, pre-miR is transported into the cell cytoplasm, and then cleavaged into the single stranded miRNA by Dicer enzyme [19, 20]. Thus, the deregulation of any factors including Drosha and Dicer enzyme in the generation of miR-202 will lead to the down-regulation of miR-202. Besides, we also identified that miR-202 plays a crucial role in cell proliferation, migration and migration by directly regulating cyclin D1 in CC cells. These finding also indicated that miR-202 is implicated in the development of CC.

Afterwards, we demonstrated that the over-expression of miR-202 inhibited the expression of cyclin D1 protein by targeting its 3′UTR of cyclin D1, suggesting that cyclin D1 is indeed a direct target of miR-202. Some studies also identified cyclin D1 gene as a key signal molecule implicated in the development of different tumors [25]. In the present work, we further demonstrated that the overexpression of cyclin D1 reverses the inhibitory effects of over-expressed miR-202 on CC cancer cell proliferation, migration and invasion. According to reports, MMPs, a kind of calcium-dependent zinc-containing endopeptidases, play a crucial role in the remodeling of tissues and organs, and the degradation of extra-cellular matrix [28, 29]. Here we identified that MMP2 and MMP9 were highly expressed in CC cells, accompanied by up-regulated cyclin D1 expression. Thus, it should be inferred that miR-202 may affect cell proliferation, migration and invasion directly or indirectly via inhibition of expression of MMP2 and MMP9.

In conclusion, this work suggested that miR-202 is down-regulated in the progression and development of CC through inhibition of cyclin D1 expression, and miR-202 can be recommended as a effective tumor-suppressing miRNA. Thus, our study provides a novel and promising therapeutic target for the treatment of CC patients.

## MATERIALS AND METHODS

### Ethics statement

Human samples used in this work were obtained from the patients or their relatives with written informed consent. This study was approved by the Ethics Committee of Zhongnan Hospital of Wuhan University.

### Cell lines and samples

Human cervical cancer cell lines SiHa, Caski and HeLa were purchased from Cell Bank of the Chinese Academy of Sciences (Shanghai, People's Republic of China). All these cells were cultured in Dulbecco's Modified Eagle's Medium (DMEM; Life Technologies, Rockville, MD, USA) containing 10% fetal bovine serum (FBS; Hyclone, Logan, UT, USA) supplemented with streptomycin (100 μg/mL) and penicillin (100 U/mL) (Life Technologies, Rockville, MD, USA). Primary CC tissues and adjacent normal CC tissues (more than 3 cm away from the tumor) were collected during surgery in Zhongnan Hospital of Wuhan University. None of the patients had received chemotherapy before surgical resection.

### RNA isolation and quantitative RT-PCR

RNA isolation and quantitative RT-PCR were conducted as described previously [18]. Total RNA was extracted from samples and cell lines using the miRNeasy Mini Kit (Qiagen, Valencia, CA, USA). The miRNA Q-PCR Detection Kit (Genecopoeia, Germantown, MD, USA) can be obtained to quantify the expression level of miRNAs according to the manufacturer's instructions. The 35 cycles were carried out as follows: (95°C for 3 min, 95°C for 12 s, and 58°C for 30 s). And then the PCR amplification was carried out using TaqMan miRNA Reverse Transcription Kit (Applied Biosystems, Foster City, CA, USA) and TaqMan Human MiRNA Assay Kit (Applied Biosystems, Foster City, CA, USA), by which the expression of miR-202 and U6 can be quantified. The reaction conditions were as follows: 95°C for 30 s, followed by 48 cycles of 95°C for 5 s, 60°C for 10 s and 72°C for 30 s. Relative miR-202 expressions were calculated with normalization to U6, and GAPDH was used to as internal controls. The relative expression was calculated by the 2-ΔΔT method. And the relative expression was calculated by the ratio of U6 or GAPDH.

The following primers were used:

miR-202, forward 5′-CCTCCCAGGCTCACGAGG CT-3′ and reverse 5′-GGTGCAGGTGCACTGGTGCA-3′; U6, forward 5′-CAAAGTCAGTGCAGGTAGGCTTA-3′ and reverse 5′-AACGCTTCACGAATTTGCGT-3′; cyclin D1, forward 5′-GTAGCAGCGAGCAGCAGAGT-3′ and reverse 5′- CGGTCGTTGAGGAGGTTGG-3′; MMP2, forward 5′-CTGTGTTGTCCAGAGGCAATG-3′ and reverse 5′-TCAGGTATTGCATGTGCTAGGT-3′; MMP9, forward 5′-TTCTGCCCGGACCAAGGATA-3′ and reverse 5′-ATGCCATTCACGTCGTCCTT-3′; GAPDH, forward 5′-TTGATGGCAACAATCTCCAC-3′ and reverse 5′-CGTCCCGTAGACAAAATGGT-3′.

### Western blot analysis

Western blot analysis was conducted as described previously [15]. Total proteins were extracted from cells or samples using extraction kit in our lab, and then extracted proteins were detected using the BCA Assay Kit (Thermo Scientific, Rockford, IL, USA). Protein samples were isolated using 10% SDS-PAGE, and then proteins were transferred onto PVDF membranes (Millipore, MA, USA). The PVDF was blocked with 5% non-fat milk for 2 h, and then, PVDF was incubated overnight at 4°C with the primary antibody, including cyclin D1 (Cell Signaling Technology, 1:1000), MMP2 (Cell Signaling Technology, 1:1000), MMP9 (Cell Signaling Technology, 1:1000) and β-actin (Santa Cruz Biotechnology, 1:1000). Signals were detected using the enhanced chemiluminescence luminol reagent (PerkinElmer Inc. Boston, Mass). β-actin was used as a loading control.

### miRNA mimic transfection

miR-202 mimic (Life technologies, Shanghai, China), and corresponding negative controls miR-NC (GenePharma, Shanghai, China) were transiently transfected into cells as reported previously [18]. Cells were seeded on a 24-well plate at 10,000 cells/well. After 14 hours, cells were transfected with a miRNA mimics at a final concentration of 10 nm using Lipofectamine 2000 (Invitrogen, Carlsbad, CA, USA) according to the manufacturer's instructions. At post-transfection 48 hours, cells were harvested for western blot or qRT-PCR analyses.

### Transfection of plasmids

We used Lipofectamine 2000 transfection reagent (Invitrogen, Carlsbad, CA) to carry out transfection of plasmids. In this work, cyclin D1 cDNA without carrying its 3′UTR was inserted into pcDNA3.1(+) vector to generate the recombinant pcDNA3.1(+)-cyclin D1 plasmid. At the same time, the pcDNA3.1(+) vector acts as control. All constructs were identified for sequence correctness using direct sequencing technology (Beijing Aodingsheng Corp., Beijing China).

### Cell proliferation

As for cell proliferation assay, cells were seeded into 96-well culture plates, and then cells were cultured in DMEM medium for three days. Afterwards, CCK-8 (10 μL; ATCC; Manassas, VA) was supplemented into each well of culture plates. Subsequently, these cells will be incubated for two hours, and then the absorbance of culture plates was detected at a wave length of 450 nm.

### Transwell assays

As for transwell assays, cells were cultured in the upper chambers of Matrigel-coated wells (1:5 dilution in serum-free medium) (Corning Costar, Cambridge, MA, USA), and then we supplemented 10% serum into the lower chamber, and then cells were cultured. After 24 h, we removed all cells loafed on the upper chambers of Matrigel-coated wells, and then cells on the lower surface of the chamber were stained using 0.1% crystal violet (Sigma, St. Louis, MO), and then cells were counted.

### Luciferase reporter assay

Luciferase reporter assay were conducted as described previously [18]. We amplified the 3′-UTR sequence of cyclin D1 based on normal human genomic DNA, and then these sequences were subcloned into the pmirGLO luciferase reporter vector (Promega, WI, USA) to generate the luciferase reporter vector. After that, all cells were cultured in 24-well plates, and then transfected with wild-type (wt) or mutant (mut) 3′-UTR vectors of cyclin D1 together with miR-202 mimics using Lipofectamine 2000. After 48 h, the luciferase activity of cells was assayed based on the Dual-Luciferase Reporter Assay System (Promega, WI, USA) following the manufacturer's protocols. The firefly luciferase activities were normalized to Renilla luciferase activity. All experiments were performed in triplicate.

### Statistical analysis

Statistical analyses were conducted by using the SPSS 17.0 software. An independent samples test like U Mann Whitney was used to generate the significance of the group-between differences. *P-value*s < 0.05 were thought as significant statistically. All data are presented as the mean ± SEM.
